# Equitable access to cochlear implants: a perspective on social justice and international obligations

**DOI:** 10.3389/fpubh.2025.1672820

**Published:** 2025-11-28

**Authors:** Pilar Suazo-Díaz, Cristian Aedo-Sanchez, Gonzalo Cuéllar-Muñoz, Enzo Aguilar-Vidal

**Affiliations:** 1 Facultad de Derecho, Universidad Autónoma de Chile, Santiago, Chile; 2 Laboratorio de Audiología y Percepción Auditiva, Departamento de Tecnología Médica Facultad de Medicina, Universidad de Chile, Santiago, Chile; 3 Clinica Santa Maria, Santiago, Chile

**Keywords:** cochlear implants, hearing loss, health inequities, human rights, health policy

## Abstract

Hearing loss is one of the most prevalent sensory conditions worldwide, affecting over 1.5 billion people and is one of the main causes of disability. For individuals with severe to profound hearing loss, cochlear implants (CIs) are one of the most effective rehabilitation options as they can significantly improve speech comprehension, language development, quality of life and social inclusion. Despite their clinical value, however, access to CIs remains highly unequal between and within countries, with implantation rates disproportionately favoring wealthier nations and people with a higher socioeconomic status. Grounded in a human rights approach, this article develops a conceptual and policy-based reflection supported by a targeted review of scientific literature and international legal standards. From this standpoint, access to CIs must be recognized as an enforceable fundamental right. This recognition implies clear international obligations for states to translate their human rights commitments into tangible, inclusive national policies. Furthermore, this perspective emphasizes the ethical responsibility to ensure the real accessibility, affordability and adaptability of cochlear implants, overcoming barriers related to socioeconomic disparities, geography, race and systemic inequalities. This perspective article highlights the urgent need for comprehensive public policies based on international human rights law to ensure equitable access to cochlear implants, advocating a rights-based approach as a necessary strategy to achieve social justice, dignity and full social participation for people with hearing disabilities.

## Introduction

Hearing loss is a very common condition that can occur at any stage of life. Estimates based on the Global Burden of Disease (GBD) initiative estimate that in 2019, approximately 1.5 billion people had some degree of hearing loss, representing 20% of the world’s population (1). Hearing loss, in turn, causes disability in nearly 500 million people, equivalent to 5% of the world’s population (2). Unfortunately, the prevalence of hearing loss is on the rise. Between 1990 and 2019, the global number of *years lived with disability* attributable to hearing loss increased by 73.6%. (1) By 2050, it is estimated that hearing loss will affect 2.5 billion people worldwide, of whom approximately 700 million will require some form of intervention (1).

Hearing impairment can have various negative consequences for those who suffer from it, being associated with difficulties in communication and emotional well-being, which can contribute to the occurrence of symptoms of depression and social isolation (3–9). In recent years, it has been reported that both depression and social isolation increase the risk of developing dementia (10, 11) which would negatively impact the quality of life of individuals with hearing loss (12, 13). Hearing screening plays a fundamental role in the investigation of hearing loss. There is evidence that children with hearing loss who are diagnosed and treated early and promptly achieve better academic, cognitive, communication, language, and even socio-emotional performance. For the identification and timely intervention of childhood hearing loss, it is essential to perform a hearing screening test on newborns (14).

Among the various strategies for treating hearing loss, cochlear implants (CI) have been one of the most successful. Although these devices were initially aimed at the pediatric population, today their use extends throughout life in those for whom hearing aids do not provide satisfactory performance (15). The criteria for indicating CI also consider aspects such as the age of onset of deafness, the duration of hearing loss, and the integrity of the auditory nerve, among others, and should always be determined by a multidisciplinary team (16–18).

A cochlear implant consists of one or two microphones located in the device’s processor. The signals obtained are sent to a processor. The processor’s purpose is to encode the acquired signals and send them to a transmitter or coil (which is located on the surface of the skin in the temporo-parietal region). This processor is held in place by the magnetic attraction generated between two magnets (one located in the transmitter itself and the other in the receiver-stimulator). This transmitter emits signals that are picked up by an antenna and a receiver-stimulator, surgically placed on the surface of the skull, under the skin in the retroauricular region. The latter decodes the message and sends it to each of the electrodes, usually located inside the cochlea, thus stimulating the cochlear nerve (15). Finally, implantation can be unilateral or bilateral. There is also the concept of a “bimodal system,” where a cochlear implant is used in one ear and a hearing aid in the other ear. Finally, there are hybrid cochlear implants (electro-acoustic stimulation or electro-acoustic hearing solutions), which combine a traditional hearing aid with a cochlear implant to address a specific type of hearing loss (16).

Auditory rehabilitation using CI is extremely effective in positively impacting the auditory and communicative performance of implant recipients, especially in terms of speech comprehension, language development, and tinnitus reduction (19–21). This also improves the quality of life of implanted users (22–24).

Despite the significant benefits offered by cochlear implants, implantation rates worldwide are far from desirable, despite being a tremendously cost-effective tool. Furthermore, the distribution of these implants shows marked inequality and inequity between different countries and between different socioeconomic groups within the same countries. In general, the use of cochlear implants is more concentrated in wealthy countries and among people with higher socioeconomic status in each country (25–28). The battery of tests used to assess hearing levels includes brainstem auditory evoked potentials (assessment of the auditory pathway and neural synchrony), otoacoustic emissions (assessment of the outer hair cells), impedance testing (assessment of the middle ear), play audiometry, and conventional audiometry (evaluation of auditory sensitivity). These tests are performed during the newborn hearing screening or in a clinical hearing evaluation (which should ideally be performed 3 months of age if the hearing screening is abnormal). In children, cochlear implantation is recommended when hearing loss is bilateral and greater than 90 dB HL (assessed by auditory brainstem response). In adults, it is also recommended when hearing loss is bilateral, equal to or greater than 90 dB HL (measured by pure tone audiometry) (29, 30).

This inequality in access to CI underscores the urgent need to develop more inclusive policies. We believe that ensuring equitable access to CI and other assistive technologies is an ethical and legal imperative to comply with basic human rights principles.

This perspective article develops a theoretical essay that analyze the multidimensional inequities in access to cochlear implants from a human rights standpoint, framing access to hearing technology as a fundamental right. By doing so, we emphasize the importance of legislation and public policies that protect dignity and encourage the full social participation of people with hearing impairments. To substantiate inequities in access to cochlear implants, we conducted a brief, narrative, non-systematic review based on a targeted search in PubMed and Web of Science, chosen for their broad regional coverage and indexing quality, using the key phrases “cochlear implant access” and “cochlear implant inequity” (and related terms: equity, inequality, disparity). The time frame covered January 2005–March 2025 to minimize reliance on older evidence that may not reflect current national or regional contexts, with a focus on original, peer-reviewed research. Duplicates, editorials, letters, and conference abstracts lacking full text were excluded. Given the perspective nature of this article, the review aimed to outline the breadth of existing evidence on inequities rather than to provide an exhaustive or in-depth analysis.

### Inequality in access to cochlear implants

Despite being a tool that has proven to be very useful in the treatment of hearing loss, access to cochlear implants is quite unequal around the world. It is difficult to estimate the number of people who could potentially benefit from a cochlear implant, but if we consider the estimates from the GBD initiative, approximately 50 million people worldwide live with severe-to-profound hearing loss (1). Naturally, not all these people are candidates for cochlear implants, but a significant percentage of them are. According to estimates, between 2021 and 2022, the total number of implant recipients reached 1 million (31) which highlights a significant gap in the coverage of people with hearing loss today and the important challenge of improving access to these devices. It is important to note that the costs associated with cochlear implants are not limited to the surgical procedure itself, but also include ongoing expenses for speech therapy, auditory rehabilitation, and long-term audiological follow-up. At the same time, cost remains a major barrier to equitable access, shaping care pathways across regions. In the United States, the total cost of a cochlear implant without health insurance ranges from $40,000 to $60,000, making this technology inaccessible to many people. This economic inaccessibility has contributed to a form of medical tourism, in which families travel to countries such as India to seek more affordable implants (between $21,000 and $30,000, including travel and the device) (32).

### Inequality in access to cochlear implants between countries

In low-income countries, less than 1% of people who are candidates for a cochlear implant can access this resource (33, 34). Fagan and Tarabichi (34) highlights how developing countries face serious difficulties, including high device costs, lack of adequate medical infrastructure, and critical shortages of trained personnel, resulting in virtually non-existent coverage (34).

The World Health Organization (35) complements this view by indicating that more than 80% of people with hearing disabilities live in low- and middle-income countries. In these regions, less than 3% of potential beneficiaries receive a cochlear implant. This global report shows how the lack of adequate public policies and insufficient health investment prevents an efficient and equitable approach to the problem (35).

### Inequality within countries

There are also significant differences in access within countries, regardless of each country’s socioeconomic level, and this is influenced by the type of social security system in each country. For example, in the United States, a country with advanced healthcare systems, access to CI is considerably better than in poorer countries; however, it remains limited and unequal. Until a decade ago, it was estimated that only about 6% of eligible adults received a cochlear implant. In the child population, although access has improved substantially, only 50% of eligible children receive this intervention. This situation is highly conditioned by individual and family economic capacity (36, 37).

Socioeconomic, racial, and geographic factors within countries also significantly condition access. Specific studies indicate that individuals belonging to high socioeconomic groups have greater access to CI, while those in situations of poverty or social vulnerability face considerably greater barriers (38, 39). In addition, ethnic and racial minorities have significantly lower implantation rates due to cultural barriers, institutionalized prejudices, and limited availability of services tailored to these communities (40–42). Rurality also emerges as another determining factor that drastically limits access due to logistical difficulties, significant distances, and lack of specialized infrastructure (40, 43, 44). These logistical and geographical barriers add to the high cost of treatment and represent an additional exclusion for the inhabitants of these areas.

In developed countries such as the United States, studies such as that by (45) highlight how, even with the availability of financial and technological resources, there are marked disparities associated with private insurance policies and socioeconomic differences, particularly affecting African American and Hispanic communities, which have less access to advanced treatments such as CI (45).

### Specific regional experiences

This section provides selected country- and subregional-level examples to complement prior content. Thus, in Europe the situation also presents significant contrasts between countries. Grose et al. (46) document how countries such as Germany and Sweden have developed robust state programs that guarantee broader and more equitable access to cochlear implants. In contrast, countries such as Romania and Bulgaria face significant challenges due to limited public coverage and high dependence on the private sector, drastically limiting equity of access (46).

Reality in Latin America reflects significant specific challenges, documented in studies such as that by Emmett et al. (47). In this region, the main barriers identified include high implant costs, insufficient coverage by public health systems, and a notable shortage of medical personnel specialized in audiology. These barriers are particularly difficult to overcome for the most vulnerable sectors of the population, who face additional exclusion due to economic and geographical factors (47).

The situation in sub-Saharan African countries is particularly critical. Evidence on cochlear implants is scarce and fragmented, and what little has been published describes programs with very low volumes, concentrated in a few centers, which are subject to high costs, shortages of specialists and reliance on charitable support rather than stable public provision. Even when initiatives exist, experiences in countries such as Malawi and Nigeria show that rehabilitation and follow-up are limited, and access is restricted to those who can afford it. Even in contexts that are relatively better equipped, socioeconomic, informational and cultural barriers persist. In short, the lack of robust data itself indicates restricted access and monitoring weaknesses in the region (48–50).

Access is also shaped by age and gender in ways that this overview can only hint at. Across health systems, pediatric implantation consistently outpaces adult uptake, with adult coverage often at or below 10%, while pediatric rates are substantially higher. This reflects the long-standing prioritization of early diagnosis and intervention, and the superior cost-effectiveness of these measures in children (34, 36). By contrast, gender-specific disparities are more difficult to identify because they are influenced by socioeconomic and cultural factors such as employment, financial autonomy, care norms and referral pathways. These dimensions merit focused analysis in their own right, which is beyond the scope of this overview.

In summary, access to cochlear implants is highly unequal and critically dependent on economic, social, racial, and geographic factors both between and within countries. These inequities constitute an urgent challenge from a human rights perspective, requiring the immediate development of comprehensive and sustainable public policies that guarantee equitable access to this essential technology.

### Equitable access from a human rights perspective

The inequalities outlined above not only affect people’s health and biopsychosocial environment, but also constitute a violation of the rights established within the human rights framework (Table 1). This imposes regulatory commitments on ratifying countries, requiring them to translate legal principles into sustainable public policies that guarantee equitable and effective access to health (51–53), thereby protecting dignity and promoting social justice (54). This framework combines instruments of varying regulatory strength, from soft law, which provides guidance on state action, to hard law, which imposes legally enforceable obligations.

**Table 1:** International frameworks of human rights.

Treaty or instrument	Recommendation items	Relevant provision	Implementation / practical application	Nature and level of enforceability
Universal declaration of human rights (UDHR)	Art. 25	Recognizes the right to an adequate standard of living that ensures health and well-being, including food, housing, and medical care.	Provides the ethical and political foundation for public health policies and universal health coverage programs.	Soft law-Non-binding. A declarative document that guides national legal systems and inspired the development of subsequent hard law instruments.
International covenant on economic, social and cultural rights (ICESCR)	Art. 12	Recognizes everyone’s right to the highest attainable standard of physical and mental health, including measures for prevention, treatment, and rehabilitation.	Requires States Parties to adopt policies and allocate resources for prevention and treatment of hearing loss, as well as for the provision and coverage of hearing technologies such as cochlear implants.	Hard law – Binding. An international treaty imposing legally enforceable obligations, monitored by the Committee on Economic, Social and Cultural Rights (CESCR).
Convention on the rights of persons with disabilities (CRPD)	Art. 4	Obliges States to adopt legislative, administrative, and other measures to guarantee the rights recognized in the Convention, eliminating discrimination and ensuring access to assistive technologies.	Requires explicit inclusion of access to hearing technologies in legislation and national policies, with dedicated budgets and regulatory frameworks.	Hard law. An international treaty with enforceable obligations, periodically reviewed by the CRPD Committee.
Convention on the rights of persons with disabilities (CRPD)	Art. 9	Requires States to ensure universal accessibility in physical environments, transport, and information and communication technologies.	Implies ensuring that candidates for cochlear implantation receive accessible information, barrier-free hospitals, and assistive technologies for follow-up care.	Hard law. Compliance is assessed by the CRPD Committee and may be invoked before national or regional courts.
Convention on the rights of persons with disabilities (CRPD)	Art. 25	Recognizes the right of persons with disabilities to enjoy the highest attainable standard of health without discrimination.	Requires the provision of hearing health services, early detection programs, and accessible cochlear implant programs.	Hard law. Establishes direct obligations of provision and non-discrimination in access to health services and technologies.
Convention on the rights of persons with disabilities (CRPD)	Art. 26	Recognizes the right to habilitation and rehabilitation, including access to assistive technologies to ensure inclusion and participation in society.	Guides community inclusion through hearing rehabilitation programs and user training for cochlear implant recipients.	Hard law. Requires States to establish accessible and sustainable rehabilitation services.
Convention on the rights of persons with disabilities (CRPD)	Art. 32	Promotes international cooperation, exchange of good practices, and technology transfer.	Encourages research collaboration on cochlear implants, technology exchange, and technical and financial support for low-income countries.	Hard law. Implemented through technical cooperation and international support programs.
General comment No. 14 of the CESCR (Right to Health)	—	Defines the right to health through the AAAQ framework (Availability, Accessibility, Acceptability, and Quality), emphasizing the protection of vulnerable groups.	Guides the application of Article 12 of the ICESCR, providing specific benchmarks for public policy and compliance assessment.	Authoritative interpretation of hard law. Not binding per se but carries strong interpretive authority and is frequently cited in judicial and policy contexts.
Sustainable development goals (SDGs 3 and 10, 2030 Agenda)	—	Promote universal health coverage and reduction of inequalities, including accessibility to health-related technologies.	Strengthen national commitments to equity, accessibility, and financing for hearing technologies.	Soft law. Global political commitments that guide policies and monitoring through voluntary indicators.

The human rights perspective is grounded in the concept of human dignity and the conditions necessary for a life of dignity. These form the foundation of the international human rights protection system (55, 56) (Caulfield, 2006). This notion is not merely abstract, but is embodied in international treaties which, once ratified, oblige States to guarantee conditions of equity for their citizens, including specific measures for the promotion and protection of health (51).

In this regard, the 1948 Universal Declaration of Human Rights (57, 58) marked a milestone by proclaiming that “everyone has the right to a standard of living adequate for the health and well-being of himself and his family, including medical care.” This principle laid the foundation for the subsequent development of more specific international treaties, such as the International Covenant on Economic, Social and Cultural Rights (ICESCR 1966), whose Article 12 recognizes the right of everyone to enjoy the highest attainable standard of physical and mental health.

The evolution of the right to health has broadened its scope, going beyond the mere absence of disease (59, 60) to include the availability, accessibility, acceptability, and quality of health services (61). This is especially relevant for persons with disabilities (62), as it guarantees their access to adequate and affordable health services, an essential condition for their well-being and full participation in society (63).

### Legal determinants in health and access to cochlear implants

The law acts as a structuring tool for healthcare systems (52, 64), exercising normative, regulatory, and governance functions (65). The approach based on legal determinants in health (52, 66) identifies four main determinants: translating principles into effective policies; strengthening governance through transparency and accountability; designing interventions based on evidence and equity; and building sustainable legal capacities at all levels of government (66). Each of these pillars translates into concrete legal obligations—for example, transparency entails a duty of accountability, and equity implies a requirement of reasonableness in the allocation of resources.

The interaction between international law and domestic law is essential in this process (52). Treaties such as the Convention on the Rights of Persons with Disabilities (CRPD) create binding obligations for States, while non-binding instruments, such as WHO resolutions or the Sustainable Development Goals (SDGs), although not legally binding, guide state action and establish ethical and technical standards that countries have agreed to respect (67). Both types of standards must be integrated into domestic law through laws, policies, and programs that ensure their effective implementation. Moreover, Article 4.2 of the CRPD incorporates the principle of progressive realization, while Article 32 establishes the obligation of international cooperation, recognizing that the full realization of the rights of persons with disabilities also depends on global solidarity.

The CRPD (adopted 2006; in force 2008) is a key instrument for the rights of persons with disabilities (68, 69), ensuring full enjoyment of rights and freedoms, including for those who require assistive technologies such as cochlear implants (70, 71). Article 25 expressly recognizes the right to health without discrimination (72, 73) (Pendo, 2010), which implies guaranteeing equitable access to medical services and specific technologies (74). In addition, it imposes on States the obligation to ensure the accessibility and affordability of these services, promoting equal opportunities.

Therefore, access to hearing technologies must not depend on arbitrary criteria or annual budget limits. It is imperative to establish a robust and financially sustainable legal framework that ensures comprehensive coverage, rehabilitation services, technical follow-up, and access without territorial, economic, or social discrimination (75). Guaranteeing such access is not only a legal obligation derived from international commitments (64), but also a requirement of social justice and respect for human dignity (76). From a legal standpoint, States must demonstrate the reasonableness of their policies by adopting deliberate, cost-effective, and non-regressive measures (77).

### Principles and obligations of states

Understanding access to hearing technologies as a human right requires recognizing that mere regulatory proclamation is not enough: it implies specific and enforceable duties for States. Human rights, in addition to establishing guiding principles, have a coercive character that translates into binding legal commitments (Gostin et al., 2018) (78), as demonstrated by the Convention on the Rights of Persons with Disabilities (CRPD). This is not just a set of aspirational statements, but a treaty that imposes specific obligations on states to take action. Within this framework, treaty bodies and legal scholars have identified three levels of obligation. The first level comprises immediate duties, such as prohibiting discrimination and adopting concrete measures. The second level comprises the minimum core content, which guarantees a basic standard essential to human dignity. The third level comprises the obligation of progressive realization, which requires states to continuously strive to fully implement rights over time. The International Covenant on Economic, Social and Cultural Rights establishes the duty to act using the maximum available resources, including through international cooperation when necessary. As a specialized instrument, the Convention on the Rights of Persons with Disabilities reinforces this mandate by requiring support services and assistive technologies to be accessible and available to all on an equal basis. In doing so, it establishes a legal framework combining immediate obligations with the duty of progressive implementation (79).

In this sense, equitable access to cochlear implants (CI) for people with hearing disabilities cannot be understood as an expression of state goodwill, but as a legal duty derived from a human rights perspective. Article 25 of the CRPD enshrines the right to health without discrimination, while Article 4 imposes on States the obligation to adopt legislative, administrative, and budgetary measures to ensure compliance. Likewise, the principle of accessibility (Art. 9) requires the elimination of physical, economic, technological, and communicative barriers that may hinder effective access to health.

The international regulatory framework requires inclusive public policies and the allocation of sufficient resources to realize these rights. This implies that the provision of technologies (80) such as CI must guarantee not only their existence, but also their affordability, availability, and suitability to individual needs, in line with the principles of acceptability and adaptability from a human rights perspective.

Theoretically, social rights, including health and access to medical technologies are enforceable fundamental rights. Their value lies in the real possibility of exercise, since legal freedom is meaningless if it is not accompanied by material conditions that allow for its effective exercise (81). Thus, guaranteeing access to CI is not an indefinite progressive option, but an immediate requirement of justice and compliance with the rule of law. The Committee on Economic, Social and Cultural Rights (82) has established that states must, at a minimum, ensure a basic level of rights that protect human dignity. This is stated in General Comments 3 and 14. More recently, article eight of the Optional Protocol to the Covenant introduced the standard of reasonableness. This standard requires public policies to be deliberate, concrete and non-discriminatory, and to be aimed at using available resources effectively and avoiding unjustified setbacks. It complements the minimum core by offering a dynamic legal test that links enforceability to the principles of equality and dignity.

For this reason, the CRPD imposes on States the duty to incorporate their international commitments into domestic laws, effective policies, and enforcement mechanisms. The justiciability of these rights means that their ownership is not merely declarative, but effective (83), enabling legal action in the event of omission or unjustified denial (84).

## Limitations and future directions

One limitation of our study is that we have mainly discussed inequality in access to cochlear implants in the US, Europe, and selected sub Saharan African countries. In Latin America, particularly South America, there is limited information reported on this topic, even for large cities or capitals. Relatedly, this study does not provide an exhaustive, region by region review; rather, it foregrounds an already established problem, lack of access, from a health and human rights perspective. Additionally, our brief, nonsystematic review and reliance primarily on English language sources may underrepresent evidence from non-English publications and limit comparability across countries due to heterogeneous indicators and reporting standards. As a future challenge, research must be conducted on how to adapt and harmonize local laws and regulations with international standards. In addition, there is the challenge of advancing studies on the actual applicability of these standards in clinical practice, financing, and service organization, so that effective equitable access to cochlear implants can be achieved (85–87).

## Conclusion

Cochlear implants are an effective and cost-efficient technology for addressing hearing impairment. However, access to them remains unequal. Many people who could benefit from this treatment do not have access to it, particularly in low- and middle-income countries where economic, geographical and social barriers are more significant.

This represents a serious shortcoming of healthcare systems and raises significant human rights concerns. Access to hearing technologies, such as cochlear implants, should be recognized not as a privilege conditioned by resource availability, but as a right that warrants progressive realization and legal protection. The Convention on the Rights of Persons with Disabilities outlines specific obligations for states in this domain, including both immediate duties and long-term commitments.

Ensuring equitable access requires sustained public policies with adequate funding, universal coverage, the elimination of structural barriers and technical monitoring. This involves integrating international commitments into domestic legislation with a focus on accessibility, affordability and adaptability.

Reducing the gap in access to cochlear implants is both urgent and possible. Failure to do so perpetuates the exclusion of a significant group of people. Recognizing the right to health requires moving toward systems that ensure timely, equitable and non-discriminatory hearing care.

## Data Availability

The original contributions presented in the study are included in the article/supplementary material, further inquiries can be directed to the corresponding author.

## References

[1] Haile LM Kamenov K Briant PS Orji AU. Hearing loss prevalence and years lived with disability, 1990–2019: findings from the global burden of disease study 2019. Lancet. (2021) 397:996–1009. doi: 10.1016/S0140-6736(21)00516-X, PMID: 33714390 PMC7960691

[2] Davis AC Hoffman HJ. Hearing loss: rising prevalence and impact. Bull World Health Organ. (2019) 97:646–646A. doi: 10.2471/BLT.19.224683, PMID: 31656325 PMC6796666

[3] Chen F Chen Y Jiang X Li X Ning H Hu M . Impact of hearing loss on cognitive function in community-dwelling older adults: serial mediation of self-rated health and depressive anxiety symptoms. Front Aging Neurosci. (2023) 15:1297622. doi: 10.3389/fnagi.2023.1297622, PMID: 38155735 PMC10753014

[4] Choi Y Go J Chung JW. Association between hearing level and mental health and quality of life in adults aged &gt;40 years. J Audiol Otol. (2024) 28:52–8. doi: 10.7874/jao.2023.00213, PMID: 37953515 PMC10808385

[5] Harianawala J Galster J Hornsby B. Psychometric comparison of the hearing in noise test and the American English matrix test. J Am Acad Audiol. (2019) 30:315–26. doi: 10.3766/jaaa.17112, PMID: 30461418

[6] Jaiyeola MT Adeyemo AA. Quality of life of deaf and hard of hearing students in Ibadan metropolis, Nigeria. PLoS One. (2018) 13:e0190130. doi: 10.1371/journal.pone.0190130, PMID: 29293560 PMC5749760

[7] Jayakody DMP Wishart J Stegeman I Eikelboom R Moyle TC Yiannos JM . Is there an association between untreated hearing loss and psychosocial outcomes? Front Aging Neurosci. (2022) 14:868673. doi: 10.3389/fnagi.2022.868673, PMID: 35663574 PMC9162786

[8] Monzani D Galeazzi GM Genovese E Marrara A Martini A. Psychological profile and social behaviour of working adults with mild or moderate hearing loss. Acta Otorhinolaryngol Ital. (2008) 28:61–6. 18669069 PMC2644978

[9] Shukla A Harper M Pedersen E Goman A Suen JJ Price C . Hearing loss, loneliness, and social isolation: a systematic review. Otolaryngol Head Neck Surg. (2020) 162:622–33. doi: 10.1177/0194599820910377, PMID: 32151193 PMC8292986

[10] Lin FR. Hearing loss and cognition among older adults in the United States. J Gerontol A Biol Sci Med Sci. (2011) 66A:1131–6. doi: 10.1093/gerona/glr115, PMID: 21768501 PMC3172566

[11] Livingston G Huntley J Sommerlad A Ames D Ballard C Banerjee S . Dementia prevention, intervention, and care: 2020 report of the lancet commission. Lancet. (2020) 396:413–46. doi: 10.1016/S0140-6736(20)30367-6, PMID: 32738937 PMC7392084

[12] Härkönen K Kivekäs I Rautiainen M Kotti V Vasama J-P. Quality of life and hearing eight years after sudden sensorineural hearing loss. Laryngoscope. (2017) 127:927–31. doi: 10.1002/lary.26133, PMID: 27328455

[13] Ronner EA Benchetrit L Levesque P Basonbul RA Cohen MS. Quality of life in children with sensorineural hearing loss. Otolaryngol Head Neck Surg. (2020) 162:129–36. doi: 10.1177/0194599819886122, PMID: 31684823

[14] Yoshinaga-Itano C. From screening to early identification and intervention: discovering predictors to successful outcomes for children with significant hearing loss. J Deaf Stud Deaf Educ. (2003) 8:11–30. doi: 10.1093/DEAFED/8.1.11, PMID: 15448044

[15] Manrique M Ramos Á de Paula Vernetta C Gil-Carcedo E Lassaletta L Sanchez-Cuadrado I . Guideline on cochlear implants. Acta Otorrinolaringol Esp. (2019) 70:47–54. doi: 10.1016/J.OTORRI.2017.10.007, PMID: 29598832

[16] Messersmith JJ Entwisle L Warren S Scott M. Clinical practice guidelines: cochlear implants. J Am Acad Audiol. (2019) 30:827–44. doi: 10.3766/JAAA.19088/ID/JR00827-31/BIB 31823835

[17] Van de Heyning P Gavilán J Godey B Hagen R Hagr A Kameswaran M . Worldwide variation in Cochlear implant candidacy. J Int Advan Otol. (2022) 18:196–202. doi: 10.5152/iao.2022.21470, PMID: 35608486 PMC10682809

[18] Zeitler DM Prentiss SM Sydlowski SA Dunn CC. American Cochlear implant Alliance task force: recommendations for determining Cochlear implant candidacy in adults. Laryngoscope. (2024) 134:S1–S14. doi: 10.1002/lary.30879, PMID: 37435829 PMC10914083

[19] Brown KD Balkany TJ. Benefits of bilateral cochlear implantation: a review. Curr Opin Otolaryngol Head Neck Surg. (2007) 15:315–8. doi: 10.1097/MOO.0b013e3282ef3d3e, PMID: 17823546

[20] Galvin JJ Fu Q-J Wilkinson EP Mills D Hagan SC Lupo JE . Benefits of Cochlear implantation for single-sided deafness: data from the house clinic-University of Southern California-University of California, Los Angeles clinical trial. Ear Hear. (2019) 40:766–81. doi: 10.1097/AUD.0000000000000671, PMID: 30358655

[21] Sharma SD Cushing SL Papsin BC Gordon KA. Hearing and speech benefits of cochlear implantation in children: a review of the literature. Int J Pediatr Otorhinolaryngol. (2020) 133:109984. doi: 10.1016/j.ijporl.2020.109984, PMID: 32203759

[22] Brodie A Smith B Ray J. The impact of rehabilitation on quality of life after hearing loss: a systematic review. Eur Arch Otorrinolaringol. (2018) 275:2435–40. doi: 10.1007/s00405-018-5100-7, PMID: 30159730 PMC6132942

[23] Rostkowska J Skarzynski PH Kobosko J Gos E Skarzynski H. Health-related quality of life in adults with profound postlingual hearing loss before and after cochlear implantation. Eur Arch Otorrinolaringol. (2021) 278:3393–9. doi: 10.1007/s00405-021-06866-7, PMID: 34101007 PMC8328909

[24] Sladen DP Carlson ML Dowling BP Olund AP DeJong MD Breneman A . Cochlear implantation in adults with asymmetric hearing loss: speech recognition in quiet and in noise, and health related quality of life. Otol Neurotol. (2018) 39:576–81. doi: 10.1097/MAO.0000000000001763, PMID: 29683995

[25] Crowson MG Semenov YR Tucci DL Niparko JK. Quality of life and cost-effectiveness of cochlear implants: a narrative review. Audiol Neurotol. (2017) 22:236–58. doi: 10.1159/000481767, PMID: 29262414

[26] Gatto A Tofanelli M Valentinuz G Mascherin A Costariol L Rizzo S . Cochlear implant cost analysis in adults: a European narrative review. Eur Arch Otorrinolaringol. (2024) 281:4455–71. doi: 10.1007/s00405-024-08591-3, PMID: 38520534 PMC11393020

[27] Neve OM Boerman JA van den Hout WB Briaire JJ van Benthem PPG Frijns JHM. Cost-benefit analysis of Cochlear implants: a societal perspective. Ear Hear. (2021) 42:1338–50. doi: 10.1097/AUD.0000000000001021, PMID: 33675588 PMC8378541

[28] Qiu J Yu C Ariyaratne TV Foteff C Ke Z Sun Y . Cost-effectiveness of pediatric Cochlear implantation in rural China. Otol Neurotol. (2017) 38:e75–84. doi: 10.1097/MAO.0000000000001389, PMID: 28379918 PMC5470857

[29] Warner-Czyz AD Roland JT Jr Thomas D Uhler K Zombek L. American cochlear implant alliance task force guidelines for determining cochlear implant candidacy in children. Ear Hear. (2022) 43:268–82. doi: 10.1097/AUD.0000000000001087, PMID: 35213891 PMC8862774

[30] Wilson K Ambler M Hanvey K Jenkins M Jiang D Maggs J . Cochlear implant assessment and candidacy for children with partial hearing. Cochlear Implants Int. (2016) 17:66–9. doi: 10.1080/14670100.2016.1152014 26913562

[31] Zeng F-G. Celebrating the one millionth cochlear implant. JASA Express Lett. (2022) 2:77201. doi: 10.1121/10.0012825, PMID: 36154048

[32] Aldè M Ambrosetti U Barozzi S Aldè S. The ongoing challenges of hearing loss: stigma, socio-cultural differences, and accessibility barriers. Audiol Res. (2025) 15:46. doi: 10.3390/AUDIOLRES15030046, PMID: 40407660 PMC12101339

[33] Bodington E Saeed SR Smith MCF Stocks NG Morse RP. A narrative review of the logistic and economic feasibility of cochlear implants in lower-income countries. Cochlear Implants Int. (2020) 22:7–16. doi: 10.1080/14670100.2020.1793070, PMID: 32674683

[34] Fagan JJ Tarabichi M. Cochlear implants in developing countries: practical and ethical considerations. Curr Opin Otolaryngol Head Neck Surg. (2018) 26:188–9. doi: 10.1097/MOO.0000000000000457, PMID: 29553960

[35] World Health Organization. Challenges facing ear and hearing care In: World report on hearing (2021). 139–98.

[36] Sorkin DL. Cochlear implantation in the world’s largest medical device market: utilization and awareness of cochlear implants in the United States. Cochlear Implants Int. (2013) 14:S12–S4. doi: 10.1179/1467010013Z.00000000076, PMID: 23453146 PMC3663290

[37] Zhang L Ding AS Xie DX Creighton FX. Understanding public perceptions regarding cochlear implant surgery in adults. Otol Neurotol. (2022) 43:E331–6. doi: 10.1097/MAO.0000000000003439, PMID: 35147605 PMC10368452

[38] Dettman S Choo D Dowell R. Barriers to early cochlear implantation. Int J Audiol. (2016) 55:S64–76. doi: 10.1080/14992027.2016.1174890, PMID: 27139125

[39] Stern RE Yueh B Lewis C Norton S Sie KCY. Recent epidemiology of pediatric cochlear implantation in the United States: disparity among children of different ethnicity and socioeconomic status. Laryngoscope. (2005) 115:125–31. doi: 10.1097/01.MLG.0000150698.61624.3C, PMID: 15630380

[40] Bush ML Burton M Loan A Shinn JB. Timing discrepancies of early intervention hearing services in urban and rural cochlear implant recipients. Otol Neurotol. (2013) 34:1630–5. doi: 10.1097/MAO.0B013E31829E83AD, PMID: 24136305 PMC3830638

[41] Raine C Atkinson H Strachan DR Martin JM. Access to cochlear implants: time to reflect. Cochlear Implants Int. (2016) 17:42–6. doi: 10.1080/14670100.2016.1155808 27099110

[42] Tolisano AM Schauwecker N Baumgart B Whitson J Kutz JW Isaacson B . Identifying disadvantaged groups for cochlear implantation: demographics from a large cochlear implant program. Ann Otol Rhinol Laryngol. (2020) 129:347–54. doi: 10.1177/0003489419888232, PMID: 31735055

[43] Cheung LL Fowler A Hassarati RT Birman CS. Distance and socioeconomic status as barriers to cochlear implantation. Otol Neurotol. (2023) 44:134–40. doi: 10.1097/MAO.0000000000003765 36624590

[44] Noblitt B Alfonso KP Adkins M Bush ML. Barriers to rehabilitation care in pediatric cochlear implant recipients. Otol Neurotol. (2018) 39:e307–13. doi: 10.1097/MAO.0000000000001777, PMID: 29649039 PMC5940514

[45] Garland JH Kennedy DG Jamil TL Patel AA Levi JR Naimi B. Socioeconomic disparities in receipt of cochlear implantation surgery in American children in 2016. Int J Pediatr Otorhinolaryngol. (2025) 194:112319. doi: 10.1016/j.ijporl.2025.112319, PMID: 40367752

[46] Grose JH Buss E Hall JW. Loud music exposure and cochlear synaptopathy in young adults: isolated auditory brainstem response effects but no perceptual consequences. Trends Hear. (2017) 21:2331216517737417. doi: 10.1177/2331216517737417, PMID: 29105620 PMC5676494

[47] Emmett SD Tucci DL Bento RF Garcia JM Juman S Chiossone-Kerdel JA . Moving beyond GDP: cost effectiveness of cochlear implantation and deaf education in Latin America. Otol Neurotol. (2016) 37:1040–8. doi: 10.1097/MAO.0000000000001148, PMID: 27518131

[48] Evangelista S. Khoza-Shangase K. Bent J., (2025). Cochlear implant decisions in South Africa: parental views, barriers, and influences. Healthcare (Basel, Switzerland), 13:787. doi: 10.3390/healthcare13070787 40218084 PMC11988570

[49] Jesuyajolu D Obuh O Edeh E. Overcoming developing-world challenges in cochlear implantation: a Nigerian perspective. Ann Med Surg. (2023) 85:5533–7. doi: 10.1097/ms9.0000000000001318, PMID: 37915666 PMC10617806

[50] Mulwafu W Chabuluka C Anderson I Strachan DR Raine CH. Development of a cochlear implant program in Malawi: progress and challenges. Cochlear Implants Int. (2024) 25:339–43. doi: 10.1080/14670100.2024.2316463, PMID: 39702988

[51] Gostin LO Wiley LF. Public health law: Power, duty, restraint. 3rd ed. California (USA): University of California Press (2016).

[52] Gostin LO Monahan JT Kaldor J DeBartolo M Friedman EA Gottschalk K . The legal determinants of health: harnessing the power of law for global health and sustainable development. Lancet. (2019) 393:1857–910. doi: 10.1016/S0140-6736(19)30233-8, PMID: 31053306 PMC7159296

[53] Hathaway O.A., (2002). Articles do human rights treaties make a difference? Yale Law Journal, Boston: Univ. School of Law Working Paper No. 02-03. (2002). Available at: https://ssrn.com/abstract=311359

[54] Wiley LF. Health law as social justice. Cornell J Law Public Policy. (2014) 24:47–106. 26812890

[55] Le Moli G. Human dignity in international law. Cambridge: Cambridge University Press (2021).

[56] Vatter M. Dignity and the foundation of human rights: toward an Averroist genealogy. Polit Relig. (2020) 13:304–32. doi: 10.1017/S1755048320000115

[57] Glendon MA. Knowing the universal declaration of human rights. Notre Dame Law Rev. (1997) 73:1153–91.

[58] McNeilly K. If only for a day’: the universal declaration of human rights, anniversary commemoration and international human rights law. Hum Rights Law Rev. (2023) 23:1–26. doi: 10.1093/HRLR/NGAD003

[59] Leary VA. The right to health in international human rights law. Health Hum Rights. (1994) 1:24–56. 10395710

[60] Yamin AE. The right to health under international law and its relevance to the United States. Am J Public Health. (2005) 95:1156–61. doi: 10.2105/AJPH.2004.055111, PMID: 15933233 PMC1449334

[61] Penchansky R Thomas JW. The concept of access: definition and relationship to consumer satisfaction. Med Care. (1981) 19:127–40. 7206846 10.1097/00005650-198102000-00001

[62] Gréaux M Moro MF Kamenov K Russell AM Barrett D Cieza A. Health equity for persons with disabilities: a global scoping review on barriers and interventions in healthcare services. Int J Equity Health. (2023) 22:236. doi: 10.1186/s12939-023-02035-w, PMID: 37957602 PMC10644565

[63] Wolbring G Deloria R. Health equity and health inequity of disabled people: a scoping review. Sustainability. (2024) 16:7143. doi: 10.3390/SU16167143

[64] Meier BM Huffstetler H Gostin LO. Human rights in global health governance. Health Hum Rights. (2018) 20:1–12.30568397

[65] Gostin LO. World health law: toward a new conception of global health governance for the 21st century. Yale J Health Policy Law Ethics. (2005) 5:413–24. 15742585

[66] Gostin LO. The legal determinants of health: how can we achieve universal health coverage and what does it mean? Int J Health Policy Manag. (2020) 10:1. doi: 10.34172/ijhpm.2020.01 PMC7947700 32610734

[67] Ghebreyesus TA. All roads lead to universal health coverage. Lancet Glob Health. (2017) 5:e839–40. doi: 10.1016/S2214-109X(17)30295-4, PMID: 28728920

[68] Degener T. Disability in a human rights context. Laws. (2016) 5:35. doi: 10.3390/laws5030035

[69] Guide T. The convention on the rights of persons with disabilities. New York and Geneva: United Nations Human Rights (2014).

[70] Mladenov T. The UN convention on the rights of persons with disabilities and its interpretation. Alter. (2013) 7:69–82. doi: 10.1016/J.ALTER.2012.08.010

[71] Stramondo JA. The right to assistive technology. Theor Med Bioeth. (2020) 41:247–71. doi: 10.1007/s11017-020-09519-4 33025313

[72] Bartlett P. Beyond the liberal subject: challenges in interpreting the CRPD, and the CRPD’S challenges to human rights. Hum Rights Law Rev. (2024) 25:ngaf005. doi: 10.1093/hrlr/ngaf005

[73] Yang DW. Disability discrimination in the provision of health insurance: article 25(e) of the UN convention on the rights of persons with disabilities. Int J Discrimination Law. (2024) 24:48–66. doi: 10.1177/13582291231204189

[74] Botelho FHF. Accessibility to digital technology: virtual barriers, real opportunities. Assist Technol. (2021) 33:27–34. doi: 10.1080/10400435.2021.1945705, PMID: 34951832

[75] Hunt P. Yamin A.E. Bustreo F. Making the case: what is the evidence of impact of applying human rights-based approaches to health? Health and Human Rights Journal, (2015) 17. Available at: https://ssrn.com/abstract=3795599 26766851

[76] Ruger JP. Health and social justice. Lancet. (2004) 364:1075–80. doi: 10.1016/S0140-6736(04)17064-5, PMID: 15380968 PMC4006198

[77] Porter B. Reasonableness and article 8(4) In: Langford M Porter B Brown R Rossi J, editors. The optional protocol to the international covenant on economic, social and cultural rights: a commentary (forthcoming) (2014). doi: 10.2139/ssrn.2481712

[78] Gruskin S. Rights-Based Approaches to Health: Something for Everyone. Health and Human Rights, (2006) 9, 5–9. doi: 10.2307/4065399 17265752

[79] Broderick A. Harmonisation and cross-fertilisation of socio-economic rights in the human rights treaty bodies: disability and the reasonableness review case study. Laws. (2016) 5:38. doi: 10.3390/laws5040038

[80] Smith EM Huff S Wescott H Daniel R Ebuenyi ID O’Donnell J . Assistive technologies are central to the realization of the convention on the rights of persons with disabilities. Disabil Rehabil Assist Technol. (2024) 19:486–91. doi: 10.1080/17483107.2022.2099987 35900971

[81] Alexy R. A theory of constitutional rights. Oxford: Oxford University Press (2010).

[82] Committee on Economic, Social and Cultural Rights (2000). General comment 14: The right to the highest attainable standard of health (twenty-second session, 2000), U.N. Doc. E/C.12/2000/4. Reprinted in compilation of general comments and general recommendations adopted by human rights treaty bodies, U.N. Doc. HRI/GEN/1/rev.6 at 85 (2003)

[83] Forman L. Justice and justiciability: advancing solidarity and justice through south Africans’ right to health jurisprudence. Med Law. (2008) 27:661–83. 19004388

[84] Müller A. The minimum core approach to the right to health In: Bielefeldt H Frewer A, editors. Menschenrechte in der Medizin / Human rights in healthcare, vol. 4. Bielefeld (Germany): Mentis Verlag (2017). 55–74.

[85] Broderick A. The long and winding road to equality and inclusion for persons with disabilities: The United Nations convention on the rights of persons with disabilities. Cambridge (United Kingdom): Cambridge University Press (2015).

[86] Forman L. What constitutes “reasonable” state action on the core obligations? Considering a right to health framework to provide essential medicines. Health Hum Rights. (2011) 13:35–48.

[87] Forman L Caraoshi L Chapman AR Lamprea E. Conceptualising minimum core obligations under the right to health: how should we define and implement the ‘morality of the depths’? Int J Hum Rights. (2016) 20:1264–81. doi: 10.1080/13642987.2015.112813

